# The current status of malaria epidemiology in Bolifamba, atypical Cameroonian rainforest zone: an assessment of intervention strategies and seasonal variations

**DOI:** 10.1186/s12889-015-2463-1

**Published:** 2015-11-06

**Authors:** Raymond Babila Nyasa, Denis Zofou, Helen Kuokuo Kimbi, Karin Mbei Kum, Roland C. Ngu, Vincent P. K. Titanji

**Affiliations:** Biotechnology Unit, Faculty of Science, University of Buea, Buea, Cameroon; Department of Zoology and Animal Physiology, Faculty of Science, University of Buea, Buea, Cameroon; Department of Environmental Science, Faculty of Science, University of Buea, Buea, Cameroon; Mboppi Baptist Hospital, Douala, Cameroon; Cameroon Christian University, PO Box 5, Bali, NW Cameroon

**Keywords:** Duration of stay, Asymptomatic malaria, Insecticide treated bed net, Bolifamba, Cameroon, Climatic factors

## Abstract

**Background:**

Global malaria has been on the decline over the past decade due to expansion of interventions. The present study aimed at determining the current status of malaria epidemiology in the context of sustained interventions and seasonal variations in Bolifamba, which represents a typical semi-urban malaria endemic community in the Cameroonian rainforest.

**Methods:**

A monthly cross-sectional survey was carried out in Bolifamba, a multi ethnic semi-urban locality on the eastern flanks of Mt Cameroon, for a year during which blood samples were collected from participants and examined for malaria parasites by microscopy. Correlation analysis of seasonal/monthly malaria prevalence was done with weather data from Ekona, a nearby village with a meteorological station. Intervention strategy such as use of Insecticide Treated Bed Net (ITBN) and risk factors such as duration of stay in the locality, age and housing type were also investigated.

**Results:**

The results revealed a malaria prevalence of 38.3 % in the rainy season, which was significantly higher than 24.4 % observed in the dry season (P < 0.0001). A high prevalence of asymptomatic malaria which was more than double the prevalence of symptomatic malaria on a monthly basis was observed, 30.7 % vs 17.8 % in the rainy and dry season respectively (p < 0.0001) and asymptomatic malaria was significantly associated with anemia (p < 0.005). April was the peak month of malaria prevalence and coincided with peak periods of both asymptomatic and symptomatic malaria. The *Plasmodium falciparum* parasite rates in the 2- up to 10-years age group (*Pf*PR_(2–10)_) was 40.8 %. The regular use of ITBN was significantly associated with low prevalence of 31.7 % as opposed to irregular or non-usage of ITBN 38.2 % (p < 0.05). Log of parasite load was found to initially increase to 2.49 with less than 5 years of stay, and decreased gradually with increasing duration of stay in the locality (p = 0.046). Climatic factors were significantly and positively associated with monthly malaria prevalence and the strongest predictors of malaria prevalence were rainfall and minimum temperature with r values of 0.563 and 0.6 respectively.

**Conclusions:**

The study highlights the role of seasonal change in modifying malaria prevalence during the year and the beneficial effect of ITBN. It also underscores a sublime problem of asymptomatic malaria associated with anemia, and indicates that partial immunity is acquired with prolonged stay in Bolifamba. This preliminary result is the basis of ongoing work to identify the antigens involved in acquired immunity.

## Background

Malaria is a major public health problem in sub-Saharan Africa. In 2000, 173 million Africans were infected with malaria as compared to 128 million in 2013 indicating, a reduction of 26 %, thanks to expansion of malaria control interventions [1]. However Cameroon remains among the 18 countries that account for 90 % of the malaria infection in subSaharan-Africa [2]. The disease is endemic nationwide in Cameroon although the level of endemicity varies from one ecological zone to another [3]. There is paucity of data on malaria epidemiology in Cameroon. A recent study in 2013 on malaria epidemiology in Bipindi, atypical Cameroonian rain forest zone without the introduction of insecticide treated bed nets (ITBNs) showed a high prevalence of 52.7 % and 43.81 % in the rainy and dry season respectively [4]. The major malaria control measure deployed by the Cameroon Government in the fight against malaria is the use of ITBN. Bolifamba is a malaria endemic locality in the Cameroonian rain forest where ITBNs have been introduced. Studies had been carried out on the epidemiology of malaria in Bolifamba before 2004 [5, 6] when only 3 % of the sub-Saharan population at risk of malaria attack had access to ITBN [2] and none of the inhabitants of Bolifamba owned an ITBN. It is therefore necessary to re-evaluate the parameters of malaria epidemiology in Bolifamba with the recent introduction of ITBN, in an era when close to 50 % of the sub-Saharan population at risk of malaria attack now have access to ITBN [2].

Bolifamba, is a malaria endemic multi-ethnic semi-urban setting located on the eastern slope of Mount Cameroon, with a reported malaria parasite prevalence of 55.9 % and 49.5 % in the rainy and dry seasons respectively, in 2005 [4] and in 2006 the values were 50.1 % and 44.2 % respectively [5]. In these studies, malaria was found to be more significantly associated with individuals living in wooden houses as compared to those living in cement blockhouses, the presence of bushes/garbage, swamps/stagnant pools of water around habitation compared were also aggravating factors. In 2005 there was complete absence of the use of Insecticide Treated Bed Nets (ITBNs) in this community and knowledge of malaria was found to be a limiting factor in the management of the disease, coupled with the absence of an affordable near-by health center [7]. Studies in Bolifamba, which had a high endemicity *Plasmodium falciparum* parasite rate (PfPR_(2–10)_ ≥ 40 %) in 2002 [4]; from the definition in A World Malaria Map [8], led to the identification of UB05, a potential malaria antigen that induces protection [9]. Naturally acquired immunity to malaria occurs in settings of perennial intense transmission [10], which are exploited in immuno-epidemiology to identify protective malaria antigens.

Within a decade after these studies were carried out, there has been increased urbanization in Bolifamba, with a radical change from building of wooden plank houses to cement blockhouses. The population has grown from 3700 a decade ago to 10,976 at the moment (Quarterly Business Plan for the Health Bolifamba 1^st^ November 2014 to 31^st^ December 2014), with most of the bushes being cleared for construction of houses. The state sanitation company (HYSACAM) has been contracted to dispose of garbage from the community thereby improving on environmental hygiene. In 2011 the Government of Cameroon with support from Global Fund for the Fight against HIV, Malaria and Tuberculosis, distributed 12.5 million ITBNs to most households in the country, including Bolifamba [2]. Furthermore, in 2011 the government constructed a community health center in Bolifamba, which provides among other services, free diagnosis and treatment of malaria for children below five years old, coupled to the large-scale education of the community on, the early diagnosis, management and treatment of malaria [7].

The aim of the present study was to determine the current status of malaria epidemiology in the context of sustained interventions and seasonal variation in Bolifamba, which represents a typical semi-urban malaria endemic community in the Cameroonian rainforest.

## Methods

### Study site

The study was conducted in Bolifamba located on the windward side of the eastern slope of Mount Cameroon. Bolifamba is found in the South West Region of Cameroon at an altitude of 530 m above sea level, 25 km from the Atlantic Ocean, on the coordinates 247.89 ^o^N and 58.24 ^o^E, and has a population of 10,976 people. The topography is almost level; a tarred road passes across the center of the village, dividing it into equal parts and a slow moving stream (the Ndongo Stream) further dissects each half of the village diagonally. The locality experiences two major seasons, dry and rainy seasons, however conditions during the transition from one season to the next appear to be uniquely different from either season. The dry season typically runs from November to February, while the rainy season starts in March and ends in October. The walls of the houses in Bolifamba are either made of wood (plank houses) or cement block, with the former predominating. Its multi-ethnic denizens are largely engaged in petty trading and subsistence agriculture to augment their income.

### Study design

A cross-sectional survey was carried out monthly from September 2013 to August 2014 to obtain approximately a hundred randomly selected samples from subjects who had not previously been invited to take part in the study. To avoid sampling a participant twice, complete names were recorded, and the data was handled with confidentiality. The study was conducted on Sunday afternoons, when most people are at home, which is a resting day and church activities are over at this time of the day, and it involved moving from house to house, inviting participants to take part in the study. Upon enrollment, information on sex, age, duration of stay in the village, regular use of ITBNs and the type of house (wooden or cement block) in which participant lives in was obtained by face to face interview with the participant or his/her guardian, in the case of minors. In this study, occasional usage of ITBN was judged as non-usage.

### Ethical considerations

Participants were schooled on the aim and potential benefit of the study prior to obtaining their informed consent. The consents of minors were obtained from their parents or guardians. Ethical clearance for this study was obtained from the University of Buea Faculty of Health Sciences-Institutional Review Board, reference number 2013/144/UB/FHS/IRB and administrative clearance from Ministry of Public Health Regional Delegation for the South West, reference number R11/MPH/SWR/RDPH/PS/108/263. Infected participants diagnosed in this study were assisted in treatment in the health center.

### Sample collection

External body temperature was measured using a digital thermometer and a finger prick was done using a sterile disposable lancet, to obtain a blood sample for laboratory analysis. The hemoglobin level of all the participants recruited into the study in the months of June, July and August and part of May (300) were measured with ten missing data on hemoglobin status and all children who were five years and below (32), recruited into the study during this period were examined by a medical doctor for splenomegaly by palpation. Hemoglobin level was determined on the spot, using STAT Site® M^Hgb^ kit by STANBIO Laboratory, Texas, USA developed by Campbell [11] and the presence and degree of anemia was classified using WHO guidelines [12].

### Laboratory analysis

Thick blood film were made from 1185 participants, air-dried and transported to the Biotechnology Unit of the University of Buea, where they were stained with 5 % Giemsa for twenty minutes, rinsed, air dried and observed under the light microscope (UNICO G380, New Jersey, USA) at X100 objective (oil immersion). A smear was declared negative, after observing 100 high power fields and no malaria parasite was seen. Positive slides were quantified by counting number of parasites against 200 white blood cells and the parasites/μl blood calculated by assuming a leucocyte count of 8000 per microlitre [13].

### Climatic factors

Weather data of Ekona used in this study was obtained from the meteorological station of the Ministry of Transport at Ekona, a neighboring village at an altitude of 378, approximately 8 km north-east from the study site. We did not have weather data for the month of August 2014.

### Statistical analyses

The data was analyzed using IBM SPSS statistics version 20 and Epi-info version 7. Cross tabulation, Chi-square analysis, t-test, ANOVA was carried out and Pearson’s significant value was obtained for contrasting parameters. Regression analysis was carried out to investigate the association of monthly prevalence to climatic factors. Graphical presentation of the data was done using Microsoft Excel 2013.

## Results

### Study sample

A total of 1185 inhabitants of Bolifamba village were sampled during the study period (September 2013 to August 2014) and comprised 677 (57.14 %) females and 508(42.86 %) males. Missing data of less than 5 % of the total sample size was incurred for the following parameters: use of ITBN, duration of stay in the study area and febrile status (Table 1). The analyses reported herein are based on the set of data obtained.

**Table 1:** Selected characteristic of the study sample

Parameter	Participants who responded		Missing data	
	Number	Percent	Number	Percent
Use of ITBN	1134	95.7 %	51	4.3 %
Duration of stay in the study area	1147	96.8 %	38	3.2 %
Age of participant	1185	100 %	0	0 %
House type of participant	1185	100 %	0	0 %
Sex	1185	100 %	0	0 %
Febrile status	1167	98.5 %	18	1.5 %
Malaria status	1185	100 %	0	0 %

### Determination of the prevalence of malaria

**Fig. 1:**
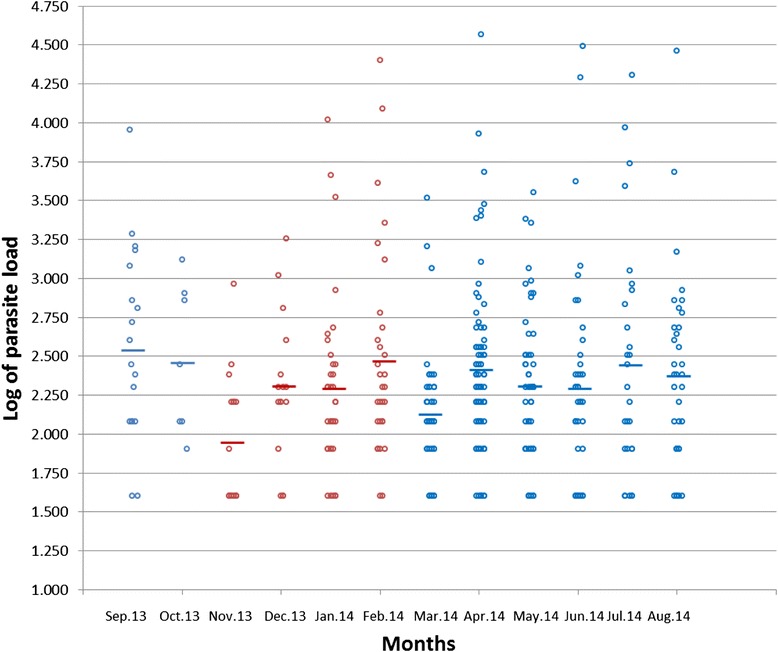
Variation of Mean Parasite load during the year. Blue = rainy season ; Red = dry season

The prevalence of malaria during the study period was 33.7 % (Table 2). Malaria was not found to be significantly associated with sex (p = 0.539) with 32.9 % (223/454) females and 34.6 % (176/332) males infected. However, malaria was found to be significantly associated with the rainy season when compared to the dry season (p < 0.0001). Of the 792 people examined in the rainy season, 303 (38.3 %) were malaria positive, as compared to 393 participants examined in the dry season, 96(24.4 %) of whom were malaria positive. Asymptomatic malaria was significantly associated with the rainy season (p < 0.0001), with a prevalence of 30.7 % (242/787) as opposed to the dry season with a prevalence of 17.8 % (70/393). However, asymptomatic malaria was not significantly associated to any age group (p = 0.095). There was no significant difference between the mean parasite load in the rainy and dry season (p = 0.337) as indicated in Fig. 1.

**Table 2:** Monthly and seasonal variation of the prevalence of malarial parasitemia and fever in Bolifamba (September 2013 –August 2014)

Season and month	Rainfall (mm)	No. and (%) of subjects			
		Examined for parasitaemia	Parasitaemic*	Examined for fever	Febrile**
Rainy Season					
September 2013	156.50	100	18 (18.0)	99	18 (18.2)
October 2013	80.00	87	7 (8.0)	86	24(27.9)
Dry Season					
November 2013	0.00	82	16 (19.5)	82	9 (10.9)
December 2013	0.00	99	16 (16.2)	98	27 (27.6)
January 2014	0.00	103	34 (33.0)	102	19 (18.6)
February 2014	0.00	109	30 (27.2)	109	22 (20.0)
Rainy season					
March 2014	36.60	91	42 (46.2)	89	11 (12.3)
April 2014	78.70	103	81 (78.6)	102	21 (20.6)
May 2014	88.50	102	51 (50.0)	100	9 (9.0)
June 2014	98.80	102	42 (41.2)	99	16 (16.2)
July 2014	197.10	99	28 (28.3)	97	13 (13.4)
August 2014	36.60	108	34 (31.5)	104	23 (22.1)
Total	772.80	1185	399 (33.7)	1167	212 (18.2)

*: χ^2^ = 175.274 (p < 0.0001); **: χ^2^ = 25.372 (p < 0.01)

**Fig. 2:**
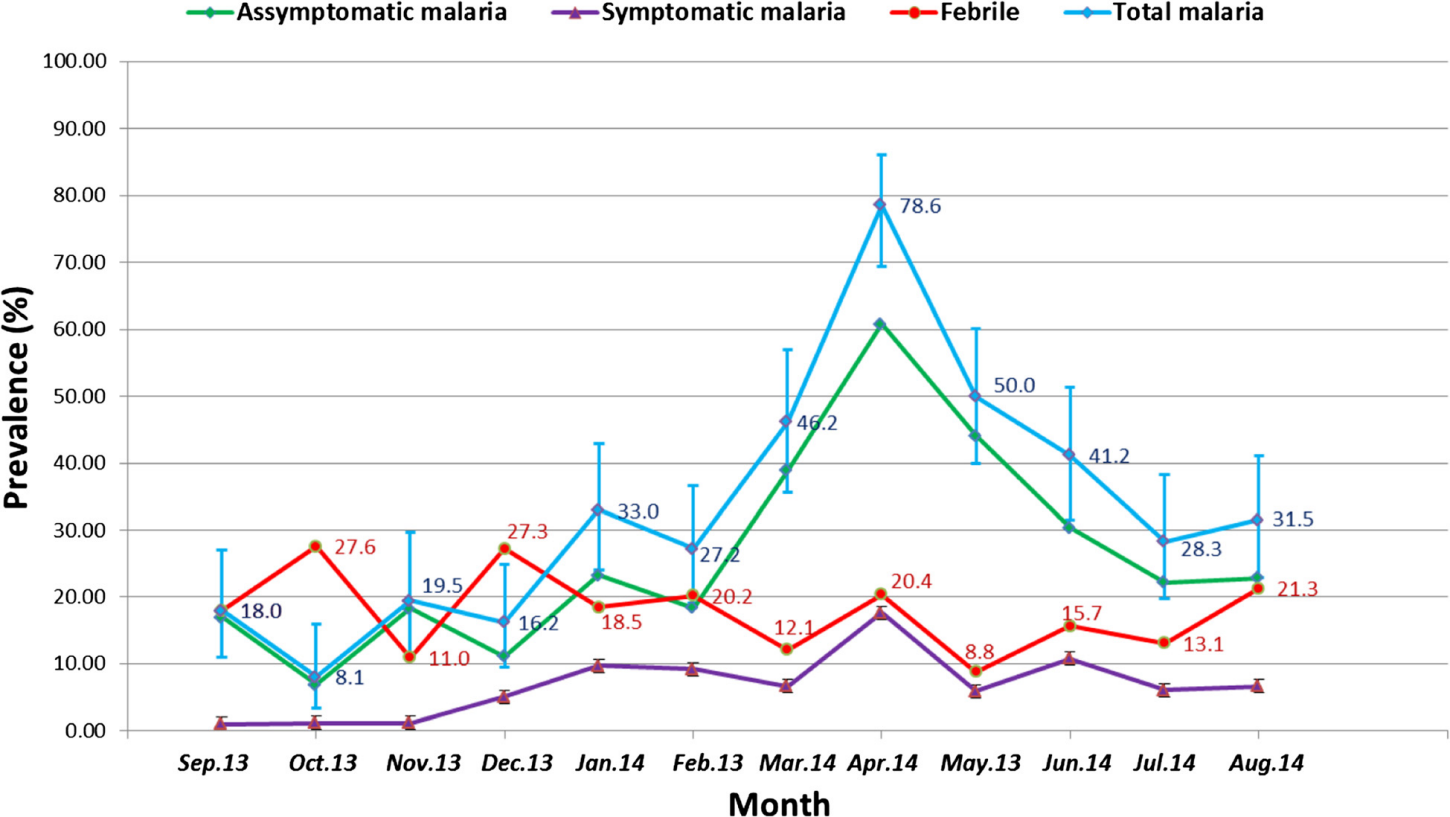
Monthly prevalence of symptomatic, asymptomatic malaria, and febrile cases in Bolifamba between September-2013 and August-2014. Asymptomatic malaria = presence of malaria parasite and temperature ≤37.5 °C, Symptomatic malaria = presence of malaria parasite and temperature >37.5 °C Total malaria = asymptomatic + symptomatic malaria

The prevalence of asymptomatic almost always more than doubled the prevalence of symptomatic malaria (Fig. 2). The peak malaria prevalence was in April with a prevalence of 78.6 (81/102) which coincided with the maximum prevalence of asymptomatic 60.8 % and symptomatic malaria (17.6 %).

### Fever

**Fig. 3:**
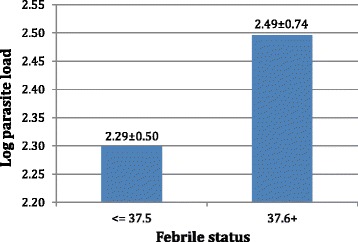
Association of mean log of parasite load with febrile status

Fever was more associated with the dry season (19.7 %) as compared to the rainy season, which had a lower prevalence of 17.4 % (p < 0.01) as seen in Table 2. Up to 90.5 % of the fever observed in April was accounted for by malaria. Fever was significantly associated with age, with the age group two to ten years having the highest prevalence of fever (Table 3). Fever was also significantly associated with a higher parasite load (p = 0.003) as shown in Fig. 3.

**Table 3:** Age-stratification of the prevalence of malaria parasitemia and prevalence of fever among Bolifamba study participants (September 2013 – August 2014)

Age group	No. and (%) of subjects				Prevalence of malaria associated with fever	
	Examined for parasitemia	Parasitemic*	Examined for fever	Febrile**	Number analyzed	Symptomatic malaria No. (%)***
<2 year (Infants)	84	22 (26.2)	81	10 (12.3)	83	3 (3.6)
[2–10 years] (Children)	387	158 (40.8)	380	93 (24.5)	385	43 (11.2)
]10-18years] (Teenagers)	212	86 (40.6)	212	36 (17.6)	212	13 (6.1)
>18 years (Adults)	502	133 (26.5)	494	73 (14.8)	500	23 (4.6)
Total	1185	399 (33.7)	1167	212 (18.2)	1180	82 (6.95)

*: χ^2^ **=** 27.07 (p < 0.01); **: χ^2^ = 16.03 (p < 0.01); ***: χ^2^ = 16.51 (p < 0.01)

### Age

**Fig. 4:**
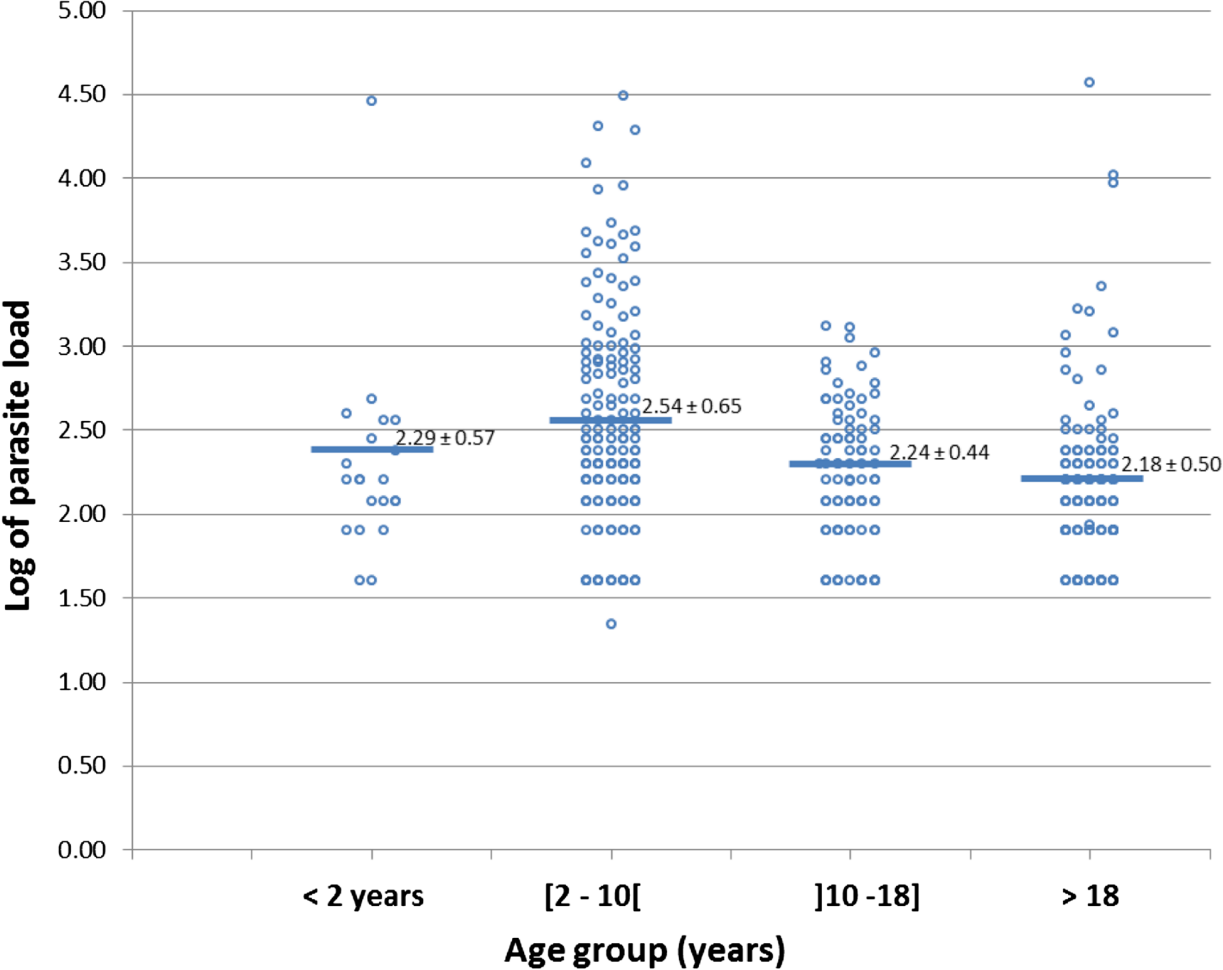
Associations of Age with mean of parasite load

Malaria prevalence was significantly associated with age (p < 0.01), with the age group 2–10 years old, having the highest prevalence of 40.6 % (86/212) as shown in Table 3. This age group also had a significantly higher parasite load (p = 0.037), than all other age groups (Fig. 4), and was different from the others by Duncan test of homogeneity. 38.7 % of the febrile cases identified in this study were associated with malaria.

### Anemia

**Fig. 5:**
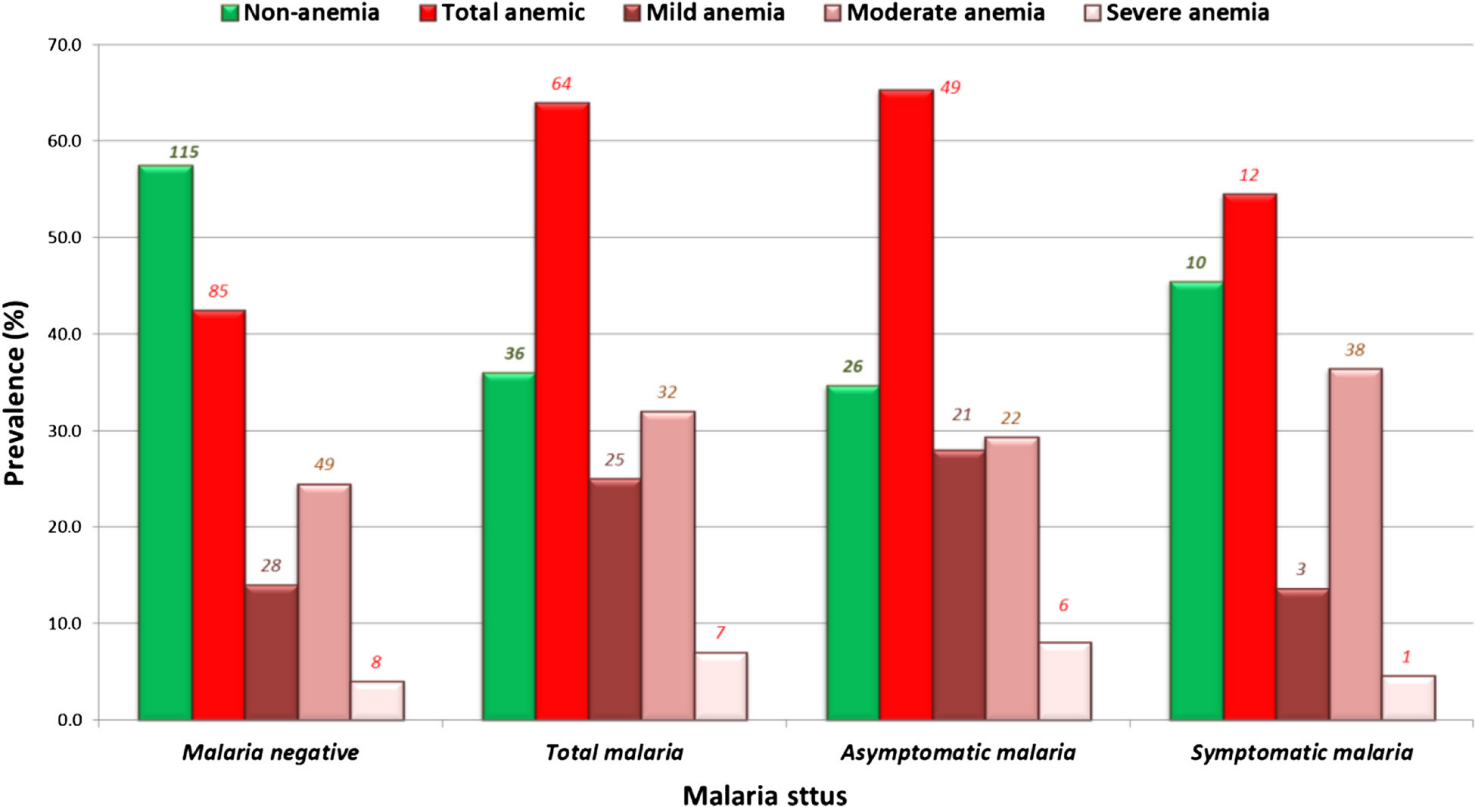
Occurrence of anemia in symptomatic, asymptomatic and malaria negative participants in Bolifamba

The prevalence of anemia was 49.7 % (149/300) and anemia was strongly associated to malaria (p < 0.001) as illustrated in Fig. 5. There was a low correlation between parasite density and anemia severity (Pearson r = 0.214; p < 0.05). Anemia occurred in both symptomatic and asymptomatic malaria. Anemia was found to be significantly associated with asymptomatic malaria (p < 0.005). Of the 32 children below five years examined for enlargement of the spleen, only a single case of splenomegaly was identified, which was not associated with malaria.

### The effect of the use of ITBNs and housing type on malaria prevalence

**Fig. 6:**
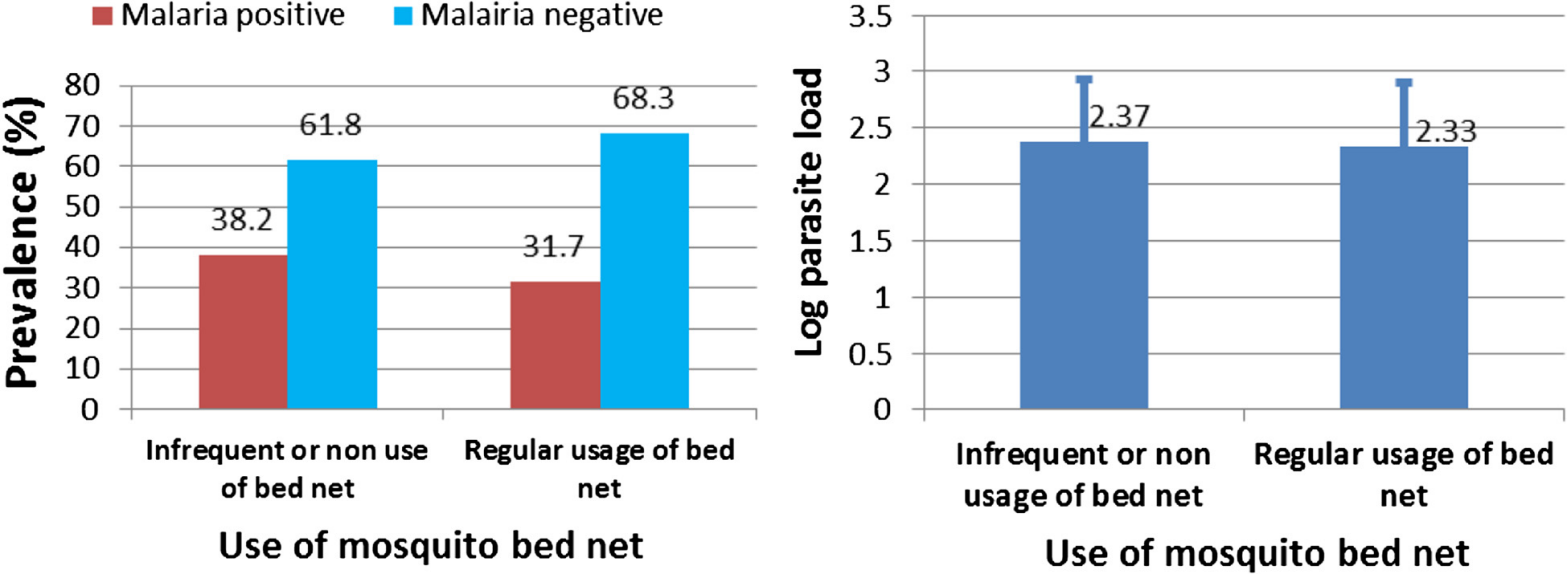
Association of malaria prevalence and log of mean parasite load with use of insecticide treated mosquito bed net

There was no association of malaria with housing type (p = 0.09) with 37 % (146/395) prevalence of malaria in blockhouse dwellers as opposed to 32 % (253/790) among wooden house occupants. Malaria was significantly associated with non-usage and irregular usage of ITBN (p < 0.05), with a 38.2 % (161/422) prevalence among ITBN users as opposed to 32 % (226/712) among regular ITBN users (Fig. 6). But there was no significant difference in parasite load between non or irregular users of ITBNs and regular users of ITBs (p = 0.79, χ^2^ = 50.97).

### Duration of stay in the study area

**Fig. 7:**
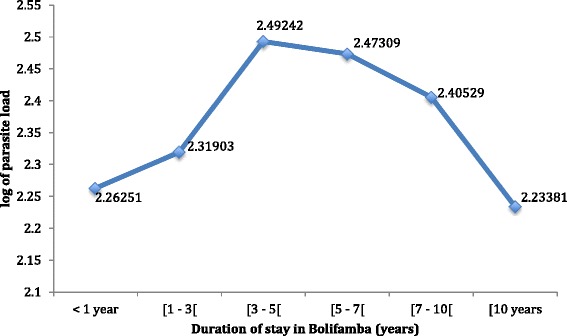
Effect of the duration of stay in Bolifamba on the malaria parasite load. <1 year: residence in the study site for less than a year; [1 – 3[ : residence for one year and more, but less than 3 years; [3 – 5[ : residence for three years and more, but less than 5 years; [5 – 7[ : residence for five years and more, but less than 7 years; [7 – 10[ : residence for seven years and more, but less than 10 years; [10: residence for 10 years and more

The prevalence data was re-plotted as a function of the duration of stay in Bolifamba (Fig. 7). The plot revealed a bell-shape with peak log of parasite load of 2.49, occurring within three to less than five years of stay in Bolifamba, there after the parasite load gradually decreased with the minimum mean log of parasite load of 2.23 in individuals who have lived for more than ten years in the locality. Analysis of variance revealed a significant difference in the mean log of parasite load (p = 0.046).

### Climatic factors

**Fig. 8:**
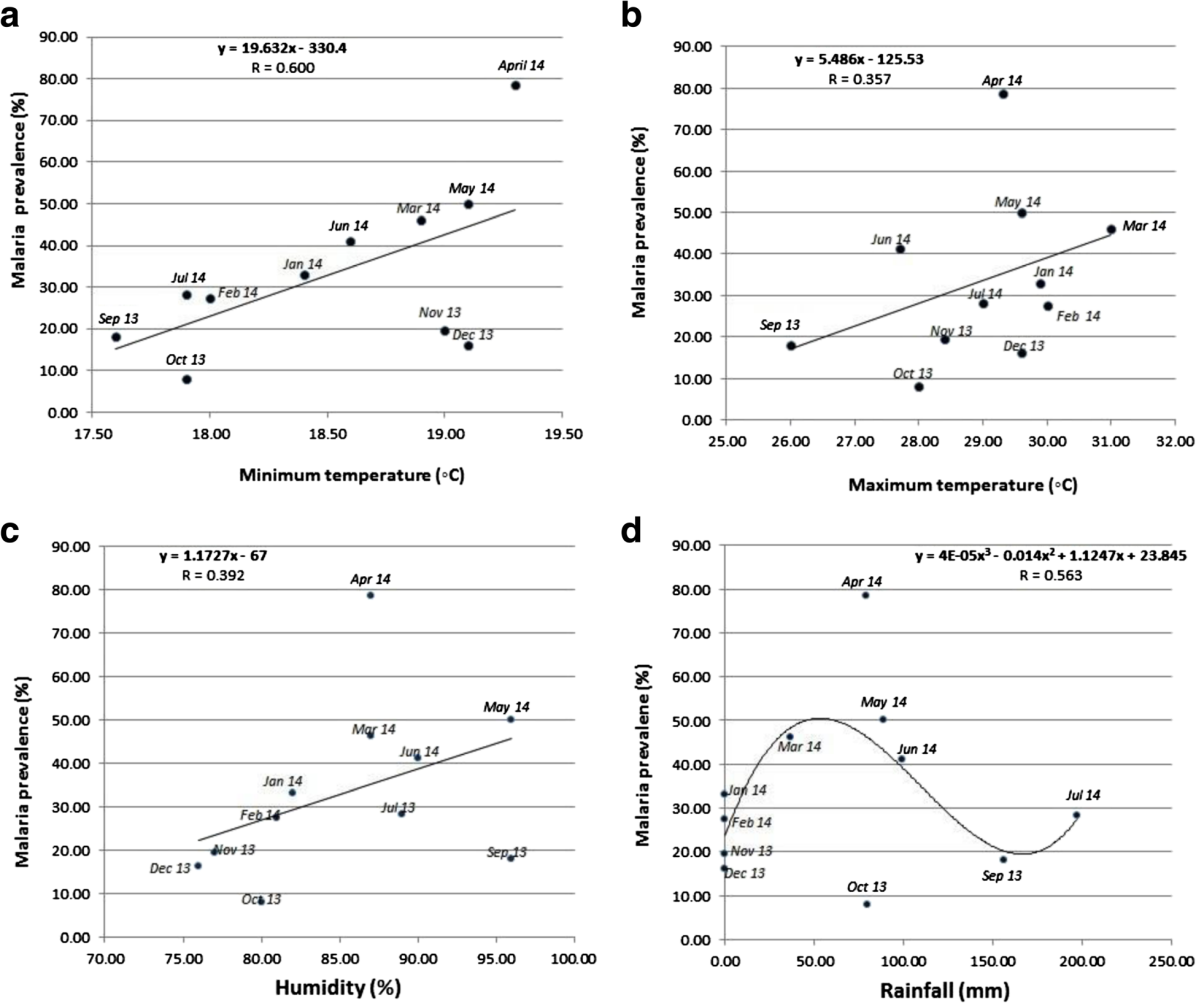
Effect of climatic factors on malaria prevalence. **a**: Correlation of malaria prevalence with minimum monthly temperatures. **b**. Correlation of malaria prevalence with maximum monthly temperatures. c: Correlation of malaria prevalence with humidity. **d**: Correlation of malaria prevalence with rainfall

The association of climatic factors and malaria prevalence was investigated using weather data from Ekona, (a village approximately 8 km from the study site) and the findings revealed that the maximum temperature, rainfall and humidity had significant positive correlations with malaria prevalence, with r values of 0.357, 0.563 and 0.392 respectively (Fig. 8). The minimum temperature had the highest correlation with malaria prevalence (r = 0.6).

### Changes in malaria prevalence over the past ten years

A comparison of the malariometric indices reported in the literature [5] and those obtained in the present study revealed significant differences. The prevalence of malaria has dropped from 52.9 % to 33.7 % but anemia prevalence has increased from 25.9 % to 49.7 %. The prevalence of malaria has also dropped in both the rainy and the dry season compared to what obtained a decade ago. Like wise the prevalence of malaria has dropped on a monthly basis but today the months of May (43.1 % to 50 %), April (54.8 % to 78.6 %) and January (29.5 % to 33 %) recorded higher malaria prevalence than ten years ago.

## Discussion

The global decrease of malaria prevalence, from intervention may abrogate naturally acquired immunity among denizens of Bolifamba. A study in Kenya showed that naturally acquired immunity to malaria is achieved by development of a threshold concentration of antibodies to malaria antigens, which occurred in an area with high endemicity (PfPR_(2–10)_ =44 %) and was absent in an area with intermediate endemicity (PfPR_(2–10)_ =29 %) [14]. However, Bolifamba is still endemic for malaria with a PfPR_(2–10)_ of 40.8 %.

The high prevalence of asymptomatic malaria corroborates findings in other malaria endemic regions [15] and the fact that asymptomatic malaria almost always more than doubled symptomatic malaria is consistent with the findings that in most malaria endemic areas, the majority of parasite carriers are asymptomatic [16]. This is a major obstacle to malaria control programs, because even sub-patent malaria is transmissible. The seasonality of malaria observed in Bolifamba with peak malaria prevalence occurring in April, at the start of the rainy season has also been reported in Accra, Ghana, although in Accra, the peak malaria months July and August, immediately follow peak rainfall in June [17].

The prevalence of malaria was highest amongst the age groups 2–10 years old (40.8 %) and the age group greater than 10–18 years. However, the parasite load was significantly higher only in the age group 2–10 years old and this age group had the highest prevalence of symptomatic malaria (11.2 %) which was significantly different from the others (p < 0.01). This finding agrees with the fact that children aged 2–10 years are the most at risk of malaria, and may also represent the main reservoir for gametocytes [18].

The high prevalence of anemia and its association with malaria (p < 0.001) strongly suggest that malaria accounts for a major part of the burden of anemia in this community. Malaria parasites feed on hemoglobin and ends up destroying red blood cells, which leads to anemia. Similar findings have been reported in Tanzania [19] and Mozambique [20]. The high prevalence of anemia was also significantly associated to asymptomatic malaria. The single case of splenomegaly observed amongst 32 children less than five years old, stood in sharp contrast to what was observed ten years ago and suggests that the multiple control interventions instituted have resulted in the reduction of malaria morbidity among children.

In this study malaria was not significantly associated with housing type (p = 0.09). The protection conferred by ITBN_S_ may have reduced the exposure of wooden house dwellers resulting in a lower prevalence than what was observed in 2006 [5]. This is presumably because mosquitoes are thought to enter wooden houses through the crevices and joints of the planks to have access to the occupants. However, their maximum biting period was between 10.00 pm-5.00 am [5], which coincides with sleeping time spent under ITBNs to limit transmission. The finding that malaria parasite prevalence was significantly associated with non-usage and/or irregular usage of ITBN compared to regular usage of ITBN (p < 0.05), is an indication that ITBN provide some level of protection against malaria and is a contributing factor to the drop in malaria prevalence from 50.1 % and 44.2 % in 2006 [5] to 38.3 % and 24.4 % in the present study, during the rainy and the dry season respectively. The association of ITBN with decreased malaria prevalence is consistent with that reported elsewhere in this region [21] and corroborates with the finding that ITBN offers some protection against malaria vectors [22, 23]. However there was no significant difference in parasite load between regular users and non users or irregular users of ITBN (p = 0.79). This implies that although ITBN plays a role in preventing infected mosquito vector from biting inhabitants of Bolifamba, when bitten by infected mosquitos, sleeping under an ITBN has no effect on the multiplication rate of the parasite within an infected person.

Naturally acquired immunity to malaria is in three stages; protection from severe disease [24], immunity to clinical symptoms, and partial protection from severe disease [25], which depends on constant transmission. Duration of stay showed an initial increase in log of parasite load up to less than 5 years of stay (2.49) and a gradual decrease in log of parasite load beyond five years of living in Bolifamba, (p = 0.046). This may be indicative of acquired immunity. It is probable that immunity to clinical symptoms is acquired within five years of living in the locality and beyond five years of stay partial protection against parasitization is gradually developed with prolonged duration of stay. This study has identified a suitable population in which protective immunity studies involving the identification and testing of malaria vaccine candidates can be undertaken, however this finding needs to be confirmed using immunological techniques.

The very high incidence of fever in October and December corresponds to the onset of the dry season and the presence of dust in the atmosphere respectively. Other pathogens in addition to malaria may be responsible for this surge in fever episodes, but the pathogens transmitted during these months are yet to be identified.

Climatic factors tend to influence the prevalence of malaria by affecting the abundance, biting habits and development of malaria parasites inside the mosquito vector. Generally minimum and maximum temperatures drop by 1 °C after every 100 m rise in altitude. The rise in altitude from intermediate to high altitude has been associated with a drop in malaria prevalence in the Mount Cameroon region [26]. Since minimum temperature is recorded in the night which corresponds to the biting time of the mosquito vector in this region [5], therefore, the decrease in temperature may lead to reduced activity and biting rate of the mosquito vector as supported by the data with an r value of 0.6 in Fig. 8. Further more, low temperatures (<15 °C) and low humidity tends to interfere with proper development of the mosquitoes in this region [27]. Rainfall had a non-linear relationship to malaria prevalence (r = 0.563). It is known that an increase in rainfall favors the accumulation of puddles, which serve as breeding sites for mosquitoes that transmit malaria parasite. But excess rainfall tends to wash off puddles with increased surface run-offs, thereby eliminating breeding sites of mosquitoes with concomitant reduction of malaria prevalence.

The equation for the measurement of malaria endemicity, predicts that in areas where the *P. falciparum* parasite rate (*Pf*PR) is 40 % or more, transmission of malaria is unlikely to be interrupted by ITBNs alone [28], and this is true for the study population. It is therefore recommended that an integrated approach be adopted in the control of malaria within this community. This should include, improved drainage system, indoor residual spraying and prophylactic treatment in the peak malaria months of March, April and May, coupled with the existing strategies in place.

## Conclusions

There is a decrease in malaria prevalence when compared with the values reported in 2005 and 2006. Regular usage of insecticide treated bed net is associated with decreased malaria prevalence in Bolifamba. However, malaria remains a serious public health threat in Bolifamba despite considerable interventions. The present study underscores a sublime problem of asymptomatic malaria associated with anemia in this locality and highlights the influence of seasonal changes, notably minimum temperature and rainfall in modifying malaria prevalence.

## Abbreviations

*Pf*PR_(2–10)_: *Plasmodium falciparum* parasite rates in the 2- up to 10-years age group
ITBN: Insecticide Treated Bed Net
